# Videotaped Patient Stories: Impact on Medical Students' Attitudes Regarding Healthcare for the Uninsured and Underinsured

**DOI:** 10.1371/journal.pone.0051827

**Published:** 2012-12-12

**Authors:** Richard Bruno, Allen Andrews, Brian Garvey, Kristin Huntoon, Rajarshi Mazumder, Jaleh Olson, David Sanders, Ilana Weinbaum, Paul Gorman

**Affiliations:** 1 Oregon Health & Science University, School of Medicine, Portland, Oregon, United States of America; 2 New York College of Osteopathic Medicine, Old Westbury, New York, United States of America; 3 OHSU Department of Medical Informatics and Clinical Epidemiology, Portland, Oregon, United States of America; University of Pittsburgh Medical Center, United States of America

## Abstract

The attitudes of medical students toward the current United States healthcare system are not well described in the literature. A graded survey was developed to assess awareness and motivation toward the care of the uninsured and underinsured as well as the impact of a video intervention on these attitudes. The survey, which showed good internal consistency (Cronbach’s alpha = 0.85), was administered before and after viewing a collection of videotaped patient stories. Although a spectrum of beliefs emerged from the analysis of survey responses, some common attitudes were identified. Eighty-five percent of respondents either agreed or strongly agreed that medical care should be provided to everyone, regardless of their ability to pay. In addition, 66% indicated they would be willing to forgo a portion of their income to provide care to those who do not have access to healthcare services. These values were strongly correlated with increasing respondent age and primary care specialty choice (p<0.01). The video intervention did not heavily influence student responses, perhaps due to a ceiling effect created by the large number of students who were already sympathetic toward the underserved. Overall, this data reflects that United States medical students recognize a need to provide care to the underserved and are willing to make personal sacrifices to meet that need.

## Introduction

In a United States policy climate dominated by the continuing debate over the Patient Protection and Affordable Care Act (PPACA), even after the Supreme Court upholding its major provisions, one of the most controversial areas is the provision of health services to the underserved. Limiting Medicaid and Medicare services and reimbursements would alter the landscape of our healthcare system far into the future, yet the voices of those who will be delivering care in that system have not been heard in these debates. In spite of the work being done by physicians and others across the nation to bring care to the underserved, tens of millions of Americans remain uninsured, [1] with nearly 45,000 unnecessary deaths every year attributable to this lack of health insurance. [2].

Attitudes among medical students toward the uninsured across the four years of medical school have been described previously. Frank, et al. found that a large percentage of medical students support universal access to health care and believe that physicians have a responsibility to care for all patients regardless of their ability to pay; however, this support was found to decline with years of medical education. [3] Another study demonstrated that medical students are more likely to be committed to caring for medically underserved patients when they enter medical school than when they graduate. [4] This might be due to the systemic barriers to care that many medical students witness during the latter two years of medical school, which occur in the clinical setting. Similarly, research on empathy demonstrates a decline throughout medical school and some authors propose engaging medical students in workshops and role-playing to maintain empathy. [5].

Others have looked at how attitudes and knowledge about the underserved affects specialty choice and allows newly practicing physicians to understand their patients’ circumstances. Weiland (2010) found that residents are not well-informed about issues concerning the underserved, including outcomes, utilization patterns, racial and ethnic disparities, and the effects of socioeconomic status on health status. [6] Wayne (2010) demonstrated that while positive attitudes toward the underserved correlated with primary care specialty choice in the past, a strong relationship no longer widely exists. [7] This research indicates that students entering medical school continue to feel that physicians have a responsibility toward individuals with limited access to health care. It also raises questions about the impact of medical education and training on student attitudes – why do these attitudes change and how can student altruism be preserved and supported?

Although Ross (2010) analyzed the potential in utilizing reflective journaling about physician responsibilities toward the underserved to help medical students wrestle with the complexities of these issues, [8] our literature search on this topic did not uncover any public health interventions which have been successful in influencing medical student commitment to the underserved. Therefore, we hypothesized that viewing filmed patient stories would impact medical students’ attitudes regarding the provision of care to underserved patients and motivate them to help meet those needs through sacrifice or advocacy.

Working from this hypothesis, we designed The VACUUM Project (Voices And Concerns of the Uninsured & Underinsured Millions) as first year medical students at the Oregon Health & Science University Our goal with the project was to assemble unfiltered video accounts of the experiences of medically underserved patients. This study aimed to (1) measure the attitudes of medical students toward the underserved, including their commitment to underserved care and their willingness to sacrifice future time and money to provide that care; and (2) evaluate the impact of a montage of videotaped patient stories on these attitudes.

## Materials and Methods

### Ethics Statement

Respondents were offered the opportunity to participate or decline to do so, and return of the anonymous survey was taken as implied consent. The study protocol, instruments, and consent procedures were approved by the OHSU IRB (IRB00006271).

This study was designed as a randomized, controlled, interventional study, with a video montage of patient stories as the intervention. Medical student volunteers in The VACUUM Project collected patient stories at community-based organizations and clinics in Portland, Oregon. Patients were invited to tell their stories and, after obtaining informed consent (see Form S1 and Form S2) for the recordings, were filmed in nearby rooms in the clinics. Selected video clips (eight patients and one provider) were edited down to a two-minute montage (see Video S1) and set to music using iMovie for the iMac (Apple Software, Cupertino, CA; music provided by Xul Solar [9]). This and other montages are available on The VACUUM Project website.

A survey instrument was developed containing twenty-two questions using a 5-point Likert scale ranging from “Strongly Agree” to “Strongly Disagree.” The questions included: demographics (4 questions), perceptions (9 questions), and degree of willingness to sacrifice (8 questions). Seven items were drawn from previously used instruments [3], [10] while eleven items were developed specifically for this project. An open-ended comment field was also included.

Subjects were recruited at nine United States medical schools (listed in Acknowledgements below), through student coordinators who were all members of the American Medical Student Association’s Health Care for All campaign steering committee (also including authors RB and KH). Each coordinator requested permission from their medical school’s Dean’s office to send an email invitation to the student body to participate in the study. A fact sheet was included with the email that explained the purpose of the study as well as contact information (see Form S3). The email contained an embedded link to the survey instrument that was hosted by SurveyMonkey.com (Portland, OR) to gain to access the survey instrument. As an incentive to participate, a $1 donation was made to Médecins Sans Frontières (Doctors Without Borders) for each completed survey.

Over the course of the study (July 12 to September 12, 2010), 895 students completed the survey. The quantitative data were analyzed using Microsoft Excel and JMP (SAS Institute, Cary, NC). Non-parametric paired analysis and univariate regression were used to analyze respondent data prior to the intervention. Descriptive statistics on all the variables and comparative analysis for selected demographic variables (i.e., age, year in school, specialty choice, and socioeconomic status) were performed. A Pearson’s chi-squared test was performed to approximate p-values for trends, and statistical significance was defined as a p-value of less than or equal to 0.05. Internal reliability of our survey was determined by Cronbach’s alpha coefficient.

One hundred and thirty-five unique responses were provided to the open-ended comment field. Qualitative data analysis was performed by identifying the major themes included in the comments and assigning each segment of each response to one of these themes. The process was used to identify experiences and attitudes shared by groups of respondents, as well as to distinguish unique opinions.

## Results

Medical students from nine medical schools were invited to participate and 895 completed the survey. Based on student coordinators’ estimates of their class size, reconciled with AAMC data, [11] the response rate was 22.7%. Of the 895 respondents, 39.8% were ages 21–24, 38.1% were ages 25–27 (see Table S1). Two fifths of respondents were in the second year, and about one fifth were in the third or fourth year of medical school. There was relatively broad representation of intended area of practice, with 38.2% of respondents planning to enter primary care, 15.1% planning on a medical specialty and 14.2% planning on a surgical career. Only (15.2%) of students reported themselves as being socio-economically disadvantaged. Four hundred and ninety-four of the 895 respondents were randomized to see the intervention video, and of these 362 randomized reported actually viewing it.

The vast majority of respondents, irrespective of demographic characteristics, specialty choice, or intervention status, agreed or strongly agreed that everyone should have access to care (96.0%) and that care should be provided regardless of ability to pay (85.4%), (see Table S2). Two thirds of respondents agreed or strongly agreed that they had an obligation to volunteer time to those without access, and two thirds expressed a willingness to forgo a portion of income to provide healthcare to the underserved. Only 1 in 5 (19.2%) of responding medical students agreed or strongly agreed that health insurance companies should remain privatized, while nearly three quarters (72.5%) agreed or strongly agreed that publicly-funded healthcare should be available to all citizens.

When analyzed by age, older respondents (25–27 and >30 years old) exhibited stronger beliefs that care should be provided regardless of ability to pay, that health insurance companies should not remain privatized, and that they would forgo pay to provide care to the underserved (p<0.01). Respondents interested in primary care demonstrated a much stronger commitment to the underserved as well, with significant differences were in the belief that everyone deserves access to care, care should be provided regardless of ability to pay, willingness to forgo pay to provide care to the underserved, and support for a national health plan (p<0.001). Associations between positive attitudes toward underserved care and advocacy were not as strong when examined by socioeconomic status or year in medical school.

Following the video intervention, a significantly higher proportion of respondents expressed that they personally wanted to be involved in providing care to those without access (83.5% of those who viewed the video vs 77.1% of those who did not, p = 0.023) and this finding was independent of age, specialty interest, socioeconomic status, or year in medical school (Table S3). Responses to the other survey questions did not vary significantly between the video intervention and control groups.

We identified seven major themes in the wide-ranging responses about experiences working with the underserved and what students would be willing to do to fulfill their role in the provision of care to the underserved:

1. How **past experiences** have affected perspectives on the underserved
2. The **future commitments** students plan to make in assisting the underserved in their careers
3. Specific ideas for **healthcare reform**
4. **Barriers** to providing care for the underserved in the future
5. **Physician and government responsibility** to provide healthcare for all
6. **Healthcare as a right** versus individual responsibility
7. Comments about the **survey instrument**

### Past Experiences

Many students mentioned previous experience working with the underserved through volunteering with free clinics, community clinics, Indian Health Services, Health Corps of AmeriCorps, county hospitals, migrant farm worker camps, and on local committees related to health services. Other students identified personal experiences, family adversity, or observing the adversity of others in the area where they grew up as a child as having affected the way they perceive care of the underserved and what they are willing to do for the underserved.

### Future Commitments

Several students expressed a commitment to work primarily in settings that provide care to the American underserved including: rural clinics, safety net clinics, community hospitals, or in doing pro bono work in these settings. Others mentioned interest in academic medicine, policy, community health outreach, or research focused on reducing disparities, or healthcare in global settings. Specialty choice was also mentioned in that many who stated their commitment to underserved patients mentioned their simultaneous commitment to primary care. Some students said they would be willing to work for reduced pay in such a setting, while others stated they could not promise to do this mostly due to concerns over student debts.

### Ideas for Reform

Many students articulated a need for systemic healthcare reform. Most of those responding were in agreement that there were a number of issues within healthcare that need to be transformed, but they did not agree on any single plan for change. Respondents proposed ideas for reform ranging from changing payment structures, reducing cost of care, incentivizing healthy behaviors, reforming health insurance, to shifting toward a socialized or single payer approach to healthcare. One respondent mentioned that real reform could only happen once society changes its values rather than simply instituting a policy change.

*I believe that health care is a right, and our country is already paying a lot for it (∼15% GDP), it's just that these dollars are not being allocated in an efficient manner. The incremental fixes that Congress is proposing will be marginally helpful, but probably almost certainly add to the deficit because it is the very fractionated nature of the health care system that costs so much.*

*I think there should be a public health insurance system, much like there is a public school system…. Patients who can afford it could still have the opportunity to pay for private insurance…*

### Barriers

Student debt and the ability to make a living were the most commonly mentioned barriers to providing care to the underserved. Other barriers mentioned by respondents included the limited number of physicians willing to care for the underserved in contrast to the enormous needs of underserved care. Another respondent offered that the underserved population is difficult to work with.

### Physician vs. Government Responsibility

While several respondents stated that physicians across all specialties have a responsibility to care for the underserved, a large number of respondents felt strongly that physicians should choose whether or not to volunteer their time serving the uninsured and should not be obligated to do so. Others took this idea further, stating that physicians already sacrifice a lot and should always be compensated for their services. There seemed to be consensus that although physicians must play a role in caring for the underserved, they should not bear the entire responsibility of doing so. Respondents were split over whether this should be the responsibility of the government or not.

### Healthcare as a Right

While a significant group of respondents felt that healthcare should be a right for all people, others felt that certain groups should be excluded or that individuals should have to take responsibility for their own health and payment for their own care. Several people stated specifically that healthcare is a right and that all people should have access regardless of their ability to pay, while others stated that healthcare should not be free for anyone and that everyone should be required to pay something. Other respondents specified that while everyone should have access to a basic level of care, those who can pay more should be able to purchase more or higher quality services. Some students clarified that specific groups, such as those who are not citizens or whose health problems are the result of unhealthy behaviors, should not receive care funded by taxpayer dollars. Several respondents also expressed concern that publicly funded care takes away personal responsibility and that people would likely take advantage of the system.

*I think all people should have to pay SOMETHING. Those who can afford to pay more should, and those that can scrape together $5 should. Paying something will, in my view and my experience, enable even the poorest of Americans to feel they've "earned" their health care. I think it will empower patients - to some small but tangible degree - to advocate for their own care and to feel freer to assert their needs and voice their concerns to their physicians.*

*I do not believe that health care is a right in so much that people can engage in unhealthy behaviors with the expectation of free and full health care. Publicly funded health care abdicates personal responsibility and accountability for one's choices.*

### Survey Instrument

Despite the fact that a few students took issue with the specific questions in the survey, a number expressed gratitude to the authors for addressing what they believe to be such an important issue. Their comments about this study’s approach seemed to suggest a deeper dedication to this topic and to a fair discourse on healthcare reform.

## Discussion

This study was designed to determine the underlying attitudes of medical students toward care of medically underserved patients and assess whether a video intervention would increase commitment to their care. Our key findings were: (1) An overwhelming majority of medical students responding agree or strongly agree that everyone should have access to healthcare (96%) and that care should be provided regardless of ability to pay (85%); (2) two thirds of medical students responding agree or strongly agree that they have the personal responsibility to volunteer time for underserved patients (67.4%) and expressed a willingness to forgo a portion of income to provide healthcare to the underserved (66.1%); (3) 72% of respondents agree or strongly agree that publicly funded healthcare should be available to all citizens; (4) these values were strongly correlated with older age and intention to pursue primary care; (5) following a video montage of patient stories, respondents were more likely to indicate a personal desire to be involved in providing care to the underserved.

In addition to these survey findings, qualitative data revealed several themes: (1) the choice to enter the medical profession was guided by a desire to help those in need; (2) commitment to the underserved is often borne out of prior personal adversity or experiences volunteering with those in need; (3) student debt was a significant barrier to deepening commitment to the underserved, due to the opportunity cost of foregone wages and personal time; (4) there is a need for reform of the current healthcare system, although there is little agreement on any single change needing to be made.

Although medical students tend to agree that all patients deserve access to care, they tend to differ in their preferences for how best to provide and pay for uncompensated care. As activist Nick Cooney has noticed, “people’s behaviors don’t match up with their attitudes enough for us to measure our success by attitudinal changes alone.” [12] Therefore, medical student action or physician sacrifice would be ideal outcomes to measure. But while medical students may lack consensus with respect to how to translate their attitudes into concrete health policy, representative groups like the American Medical Student Association and the American Medical Association – Medical Student Section offer organized advocacy days on the local and national levels to help students put those beliefs to action. Examples of advocacy in medicine date back to Rudolph Virchow’s social medicine dictum that, “Physicians are the natural attorneys for the poor, and social problems fall to a large extent within their jurisdiction,” [13] and are more recently reflected in the American Board of Internal Medicine’s Physician Charter calling for physicians to fulfill the commitment to medical professionalism by striving, “to reduce barriers to equitable health care… without concern for the self-interest of the physician or the profession.”

With the burden of future healthcare delivery falling heavily on medical students' shoulders, it will be imperative for them to continue to participate in the development of a system that accurately reflects their values, namely, support for universal access and systemic reform.

### Limitations

There are certain limitations of our study that should be considered. First, due to the fact that the questionnaire response rate was 22.7%, the generalizability of our findings depends on the extent to which the study respondents are representative of all medical students. The students that were motivated to respond could have been more interested in this issue, leading to a selection bias for students that had more strongly held positions about the uninsured than most medical students.

After analysis, we propose a “ceiling effect” as another possible limitation. Due to the fact that medical students have been shown to have a high level of empathy [2]–[4], relative differences between the intervention group and controls would be difficult to capture. Yet, this potential confounder was not possible to avoid, as medical students were the population under study. Contrary to most of the medical student empathy literature, we found that older respondents (25+) exhibited stronger beliefs that care should be provided regardless of ability to pay (p = 0.0069).

### Future Directions

Qualitative expression of the respondents’ opinions provided insight into the quantitative findings of this study. Except for increased interest in serving the underserved, this cross-sectional study was not successful in demonstrating conclusively the impact of video patient stories on medical students’ attitudes toward the underserved. We intend to follow up with other groups of more experienced medical professionals (physicians and nurses) and the general public in addition to medical students. In these populations, it will be important to address the so-called victim-denigration phenomenon, by which more innocent victims tend to be denigrated more despite seeming more deserving of sympathy. [14].

As the future healthcare environment is extremely dynamic with frequent infrastructural adjustments and major policy changes, further studies need to assess longitudinal changes in medical students’ attitudes toward the healthcare system and its shortcomings with access of care for the underserved.

This paper builds off of work done in two other studies which utilized medical student surveys of attitudes on the Patient Protection and Affordable Care Act (PPACA) [15], and during a Capitol Hill advocacy day (unpublished data), concluding that students favor healthcare reform and medical school curricula reform to include stronger training in advocacy. Collectively, these three studies point to the importance of moving forward with efforts to understand the attitudes of future healthcare providers and harnessing the level of empathy among medical students to create greater systemic change and improve care among the underserved.

## Supporting Information

Form S1 **Authorization to Use and Disclose Protected Health Information.**
(PDF)

Form S2 **Consent to Audio/Video Record.**
(DOC)

Form S3 **Fact Sheet**
(PDF)

Table S1 **Respondent demographics.**
(DOCX)

Table S2 **Demographic classifications and attitudes toward the underserved.**
(DOCX)

Table S3 **Video intervention and attitudes toward the underserved.**
(DOCX)

Video S1 **Uninsured patients’ video montage.**
(MP4)
