# Correction: Videotaped Patient Stories: Impact on Medical Students' Attitudes Regarding Healthcare for the Uninsured and Underinsured

**DOI:** 10.1371/annotation/c1378388-82f7-4470-8fc7-c148c291eb97

**Published:** 2013-06-04

**Authors:** Richard Bruno, Allen Andrews, Brian Garvey, Kristin Huntoon, Rajarshi Mazumder, Jaleh Olson, David Sanders, Ilana Weinbaum, Paul Gorman

There is an error in Supporting Information. Tables S1-S3 should not be included in the Supporting Information Section. These tables are part of the main text. The citations for Tables S1-S3 in the main text should be considered citations to Tables 1-3. 

