# Supplementary material for: Videotaped Patient Stories: Impact on Medical Students' Attitudes Regarding Healthcare for the Uninsured and Underinsured
Source: PLoS One. 2012 Dec 12;7(12):e51827. doi: 10.1371/journal.pone.0051827 (PMC3520926; doi:10.1371/journal.pone.0051827)
Supplement: Form S1 — Authorization to Use and Disclose Protected Health Information. (PDF) [file pone.0051827.s001.pdf]

OREGON
HEALTH & SCIENCE
UNIVERSITY
Hospitals and Clinics
OHSU Communications
3181 SW Sam Jackson Park Rd,
Mail Code L217
Portland, OR 97239-3098
(503) 494-8231, Fax (503) 494-8246

ACCOUNT NO.
MED. REC. NO.
NAME
BIRTHDATE

Patient Identification

AUTHORIZATION TO USE AND DISCLOSE PROTECTED HEALTH INFORMATION

*ALL SECTIONS OF THE AUTHORIZATION **MUST** BE COMPLETED OR THE AUTHORIZATION WILL NOT BE ACCEPTED.*

I authorize OHSU to use and disclose a copy of the specific health information described below regarding:
____ (Name of individual)

consisting of: _____ My name _____My age _____ My city, county or state residence _____Information about my specific injuries _____ An interview with me at OHSU _____ A photograph of me at OHSU _____ Videotape of me at OHSU _____ Other information as described here: ___________________

to OHSU for the purpose of: ____Media request (__all media __ specified media________________)
____OHSU Advertisement ____ OHSU Brochure ____OHSU Annual report ____ OHSU Magazine ____OHSU Newsletter ____ OHSU Marketing campaign ____OHSU Fund-raising campaign ____ OHSU World Wide Web site
____Other. Title of project :____.

If the information to be disclosed contains any of the types of records or information listed below, additional laws relating to the use and disclosure of the information may apply. I understand and agree that this information will be disclosed only if I place my ***initials*** in the applicable space next to the type of information.

_____ HIV/AIDS information
_____ Mental health information
_____ Genetic testing information
_____ Drug/alcohol diagnosis, treatment, or referral information

You do not need to sign this authorization. Refusal to sign the authorization will not adversely affect your ability to receive health care services or reimbursement for services. The only circumstance when refusal to sign will mean you will not receive health services is if the health services are solely for the purpose of providing health information to someone else, and the authorization is necessary to make that disclosure. Your refusal to sign this authorization does not adversely affect your enrollment in a health plan or eligibility for health benefits, unless the authorized information is necessary to determine if you are eligible to enroll in the health plan.

You may revoke this authorization in writing at any time. If you revoke your authorization, the information described above may no longer be used or disclosed for the purposes described in this written authorization. Any uses or disclosures already made with your permission cannot be undone.

To revoke this authorization, please send a written statement to Med. Correspondence, Health Information Services, OP17A, OHSU 3181 SW Sam Jackson Pk Rd. Portland, OR 97239-3098, and state that you are revoking this authorization.

I understand that the information used or disclosed pursuant to this authorization may be subject to re-disclosure and no longer be protected under federal law. However, I also understand that federal or state law may restrict re-disclosure of HIV/AIDS information, mental health information, genetic information and drug/alcohol diagnosis, treatment or referral information.

I have read this authorization and I understand it.

This authorization expires one year from the date of signing unless revoked or otherwise specified below:

(enter alternative expiration date or event) ________________________________

MR1470

By:____ Date:______________

(Signature of individual or personal representative)

Description of personal representative's authority:_________________________________
