# Supplementary material for: Videotaped Patient Stories: Impact on Medical Students' Attitudes Regarding Healthcare for the Uninsured and Underinsured
Source: PLoS One. 2012 Dec 12;7(12):e51827. doi: 10.1371/journal.pone.0051827 (PMC3520926; doi:10.1371/journal.pone.0051827)
Supplement: Form S2 — Consent to Audio/Video Record. (DOC) [file pone.0051827.s002.doc]

[TheVacuum.org](http://www.TheVacuum.org/) project
The **V**oices **A**nd **C**oncerns of the **U**ninsured & **U**nderinsured **M**illions

*“Keep patients from being swept under the rug”*

*“Help fill the void in healthcare*

CONSENT TO VIDEO/AUDIO RECORD

I, ______________________________, agree to the recording of an interview conducted by a medical student from Oregon Health and Science University (OHSU).

I understand that this recording may be played in public, on the internet, or over the radio. I also understand that the recording may be edited and parts of the interview may be rearranged or removed.

The purpose of this recording is to inform the public about the experience of being without health insurance or caring for those who do not have health insurance.

_____ By placing your initials here, your name and other identifying information will be kept strictly confidential. Please be aware, however, that your voice, likeness, and details you say may expose your identity to listeners.

Over the course of the interview, please attempt to avoid using names or other information that will specifically identify other individuals.

By signing this form, you agree to the recording and public broadcasting of this interview.

______________________________ _____________

Printed name of Interviewee/Guardian Date

______________________________ ____________

Signature of Interviewee/Guardian Date

______________________________ ________________________

Printed Name of Interviewer Signature of Interviewer
