# Supplementary material for: Videotaped Patient Stories: Impact on Medical Students' Attitudes Regarding Healthcare for the Uninsured and Underinsured
Source: PLoS One. 2012 Dec 12;7(12):e51827. doi: 10.1371/journal.pone.0051827 (PMC3520926; doi:10.1371/journal.pone.0051827)
Supplement: Form S3 — Fact Sheet (PDF) [file pone.0051827.s003.pdf]

Changes in Medical Student Attitudes Regarding Health Care for the Underserved
Fact Sheet

Purpose The purpose of this protocol is to assess medical students' attitudes regarding health care for the underserved and assess the impact of theVACUUM.org project (Voices And Concerns of the Uninsured & Underinsured Millions) video footage on these attitudes. There has not been a recent evaluation on this issue in the wake of national health care reform. Therefore, this research will provide a more detailed picture of medical students' current beliefs on this topic, and we will be able to compare with future medical student and clinician attitudes.

Methods The research questions will be answered using complementary survey methods. For Phase I of our research project, we are planning to implement a nationwide survey of 15 medical schools. The survey will be administered electronically via an online survey tool and distributed to medical students in their clinical rotation years (MS4) at selected schools via email. Phase II will be implemented via a wireless classroom response system during Oregon Health & Science University's (OHSU) Principles of Clinical Medicine course to pre-clinical students (MS1-MS2). One dollar will be donated to Médecins Sans Frontières (Doctors Without Borders; up to $1000) for every person who completes the survey.

Consent Participation in this research is entirely voluntary, and will last only as long as it takes to complete the survey (approximately 5 minutes for the online survey, and two 5 minute sessions for the in-class survey). A person may choose to participate or may refuse to participate as they wish, and may change their mind at any time, for any reason.

Confidentiality No identifying information will be collected.

Support This research is supported by the Robert Bacon scholarship from the OHSU School of Medicine.

Investigators This research is being conducted by a group of co-investigator medical students at OHSU, in conjunction with student research coordinators at fifteen US medical schools, and led by Principal Investigator, Paul Gorman, MD, OHSU School of Medicine.

Questions If you have questions or concerns about this research, please call:

Paul Gorman, MD
Oregon Health and Science University
3181 SW Sam Jackson Park Rd.
Portland, Oregon 97201
503 494-4025
gormanp@ohsu.edu
