# Supplementary material for: Videotaped Patient Stories: Impact on Medical Students' Attitudes Regarding Healthcare for the Uninsured and Underinsured
Source: PLoS One. 2012 Dec 12;7(12):e51827. doi: 10.1371/journal.pone.0051827 (PMC3520926; doi:10.1371/journal.pone.0051827)
Supplement: Table S1 — Respondent demographics. (DOCX) [file pone.0051827.s004.docx]

Table S1: Respondent demographics

|  | ***Number*** | ***percent of total (%)*** |
| --- | --- | --- |
| **Age (years)** |  |  |
| <21 | 6 | 0.7 |
| 21-24 | 356 | 39.8 |
| 25-27 | 341 | 38.1 |
| 28-30 | 110 | 12.3 |
| >30 | 82 | 9.2 |
|  |  |  |
| **Year in school** |  |  |
| MS1 | 75 | 8.4 |
| MS2 | 375 | 41.9 |
| MS3 | 169 | 18.9 |
| MS4 | 183 | 20.4 |
| MPH/PhD/Other | 93 | 10.4 |
|  |  |  |
| **Specialty interest at time of survey** | |  |
| Primary Care (Family Med, Internal Med, Peds, OB/GYN) | 342 | 38.2 |
| General Surgery | 22 | 2.5 |
| Surgical sub-specialty | 105 | 11.7 |
| Internal Medicine specialty | 135 | 15.1 |
| Other/Undecided | 291 | 32.5 |
|  |  |  |
| **Socioeconomically disadvantaged background (self report)** | | |
| yes | 136 | 15.2 |
| no | 759 | 84.8 |
|  |  |  |
| **Total** | **895** | **100** |
