# Supplementary material for: Videotaped Patient Stories: Impact on Medical Students' Attitudes Regarding Healthcare for the Uninsured and Underinsured
Source: PLoS One. 2012 Dec 12;7(12):e51827. doi: 10.1371/journal.pone.0051827 (PMC3520926; doi:10.1371/journal.pone.0051827)
Supplement: Table S2 — Demographic classifications and attitudes toward the underserved. (DOCX) [file pone.0051827.s005.docx]

Table S2. Demographic classifications and attitudes toward the underserved.

|  | **Strongly Agree** | **Agree** | **Neutral** | **Disagree** | **Strongly Disagree** | **Age** | **Year in School** | **Specialty*** | **Socioeconomic Status**** |
| --- | --- | --- | --- | --- | --- | --- | --- | --- | --- |
|  |  |  |  |  |  | *(p-value, R^2^)* | *(p-value, R^2^)* | *(p-value, R^2^)* | *(p-value, R^2^)* |
| Everyone should have access to medical care | | | | | | | | |  |
|  | 584(65.2) | 276(30.8) | 22( 2.5) | 9 (1) | 4 (0.5) | 0.621,0.009 | 0.125 ,0.016 | **0.0001,0.029** | 0.2962,0.0034 |
|  |  |  |  |  |  |  |  |  |  |
| Care should be provided regardless of ability to pay | | | | | | | | |  |
|  | 403 (45.03) | 361(40.34) | 68( 7.60) | 46(5.14) | 17 (1.90) | **0.0069,0.0162** | 0.5325,0.0072 | **0.0001,0.0274** | 0.3107,0.0023 |
|  |  |  |  |  |  |  |  |  |  |
| It is my obligation to volunteer my time to those who don’t have access | | | | | | | | |  |
|  | 204 (24.76) | 351(42.6) | 128(15.53) | 116(14.08) | 25(3.03) | 0.0679,0.0110 | 0.5136,0.0067 | **0.0001,0.0289** | 0.1440,0.0030 |
|  |  |  |  |  |  |  |  |  |  |
| I would forgo a portion of my income to provide care | | | | | | | | |  |
|  | 217 (24.25) | 375 (41.90) | 168 (18.77) | 90 ( 10.06) | 45 (5.03) | **0.0001, unreadable** | 0.0396,0.0121 | **0.0001,0.0340** | 0.3395,0.0018 |
|  |  |  |  |  |  |  |  |  |  |
| Health Insurance companies should remain privatized | | | | | | | | |  |
|  | 51 (5.7) | 121( 13.52) | 310 (34.64) | 275 (30.73) | 138 (15.42) | **0.0028,0.0139** | 0.1003,0.0091 | **0.0001,0.0221** | 0.5504,0.0012 |
|  |  |  |  |  |  |  |  |  |  |
| Publicly funded healthcare should be available to all citizens | | | | | | | | |  |
|  | 87 (9.72) | 236 (26.37) | 269 (30.06) | 194 (21.68) | 109 (12.18) | 0.8402,0.0038 | 0.1641,0.0078 | **0.0018,0.0137** | 0.0498,0.0035 |

Raw value (% of respondents)

* Primary care vs not

** Disadvantaged vs not
