# Supplementary material for: Videotaped Patient Stories: Impact on Medical Students' Attitudes Regarding Healthcare for the Uninsured and Underinsured
Source: PLoS One. 2012 Dec 12;7(12):e51827. doi: 10.1371/journal.pone.0051827 (PMC3520926; doi:10.1371/journal.pone.0051827)
Supplement: Table S3 — Video intervention and attitudes toward the underserved. (DOCX) [file pone.0051827.s006.docx]

Table S3. Video intervention and attitudes toward the underserved.

|  |  | **Strongly Agree** | **Agree** | **Neutral** | **Disagree** | **Strongly Disagree** | **p** | **R^2^** |
| --- | --- | --- | --- | --- | --- | --- | --- | --- |
|  |  |  |  |  |  |  |  |  |
| Everyone should have access to medical care | | | | | | | | |
|  | Yes* | 244 (67) | 108 (30) | 5 (1) | 4(1) | 1 (0) | 0.3788 | 0.006 |
|  | No** | 88 (67) | 40 (30) | 2 (2) | 2 (2) | 0 (0) |  |  |
|  | Control^ | 252 (63) | 128 (32) | 15 (4) | 3 (1) | 3 (1) |  |  |
|  |  |  |  |  |  |  |  |  |
| Care should be provided regardless of ability to pay | | | | | | | | |
|  | Yes | 176 (49) | 144 (40) | 21 (6) | 16 (4) | 5 (1) | 0.3519 | 0.0043 |
|  | No | 58 (44) | 53 (40) | 11 (8) | 5 (4) | 5 (4) |  |  |
|  | Control | 169 (42) | 164 (41) | 36 (9) | 25 (6) | 7 (2) |  |  |
|  |  |  |  |  |  |  |  |  |
| It is my obligation to volunteer my time to those who don’t have access | | | | | | | | |
|  | Yes | 98 (28) | 152 (43) | 51 (14) | 44 (13) | 7 (2) | 0.5234 | 0.0031 |
|  | No | 24 (22) | 46 (43) | 19 (18) | 14 (13) | 5 (5) |  |  |
|  | Control | 82 (23) | 153 (42) | 58 (16) | 58 (16) | 13 (4) |  |  |
|  |  |  |  |  |  |  |  |  |
| I would forgo a portion of my income to provide care | | | | | | | | |
|  | Yes | 92 (25) | 158 (44) | 62 (17) | 38 (11) | 12 (3) | 0.6435 | 0.0024 |
|  | No | 30 (23) | 53 (40) | 26 (20) | 15 (11) | 8 (6) |  |  |
|  | Control | 95 (24) | 164 (41) | 80 (20) | 37 (9) | 25 (6) |  |  |
|  |  |  |  |  |  |  |  |  |
| Health Insurance companies should remain privatized | | | | | | | | |
|  | Yes | 17 (5) | 47 (13) | 131 (36) | 119 (33) | 48 (13) | 0.1109 | 0.005 |
|  | No | 10 (8) | 9 (7) | 46 (35) | 42 (32) | 25 (19) |  |  |
|  | Control | 24 ( 6) | 65 (16) | 133 (33) | 114 (28) | 65 (16) |  |  |
|  |  |  |  |  |  |  |  |  |
| Publicly funded healthcare should be available to all citizens | | | | | | | | |
|  | Yes | 123 (35) | 150 (43) | 46 (13) | 21 (6) | 12 (3) | 0.1099 | 0.0059 |
|  | No | 41 (38) | 34 (31) | 20 (19) | 8 (7) | 5 (5) |  |  |
|  | Control | 104 (28) | 145 (40) | 65 (18) | 27 (7) | 23 (6) |  |  |
| I personally want to be involved in providing individuals who do not have access to health care services | | | | | | | | |
|  | Yes | 143 (17) | 151 (18) | 45 (5) | 13 (2) | 0 (0) | 0.023 | 0.009 |
|  | No | 40 (5) | 40 (5) | 17 (2) | 10 (1) | 1 (0) |  |  |
|  | Control | 124 (15) | 160 (19) | 54 (7) | 19 (2) | 7 (1) |  |  |

Raw value (% of respondents)

*Randomized to intervention group and viewed the entire video.

**Randomized to intervention group and did not view the entire video.

^Randomized to control group, did not view the entire video.

*p-value signifies difference between all three groups (Yes/No/Control)*
